# Carbolic Acid Treatment in Surgery

**Published:** 1869-03

**Authors:** 


Carbolic Acid Treatment in Surgery.—Dr. Kelburne King, of Hull, read a paper "On Carbolic Acid and the Antiseptic Treatment in Surgery." Modern science shows that suppuration is not a wearing process in cases of those wounds which do not heal by first intention; granulation can occur without suppuration. Access of air is the most common cause of suppuration, not on account of the air itself, but the germs which are contained in it. Carbolic acid diminishes or annihilates suppurative action. The author had employed in different cases dressings of (1) solution of carbolic acid; (2) carbolic oil, proportion one to four; (3) carbolic putty—i. e., whiting added to carbolic oil to a desired consistence. The author claimed for the treatment: 1. That decomposition of discharges is prevented. 2. That it enables those parts injured beyond redemption to slough away without becoming foci for the re-formation of pus. 3. That it exercises a control over the formation of pus in the center of wounds. 4. That it diminishes the chances of blood-poisoning. 5. That it is of signal service to the patient himself and to those surrounding him, as all fetor is absolutely prevented.

